# Mirror Visual Feedback Selectively Attenuates Crossover Fatigue in Distal Upper Limb Musculature: A Randomized Controlled Crossover Investigation Comparing Children and Adults

**DOI:** 10.3390/life16030435

**Published:** 2026-03-08

**Authors:** Aymen Ben Othman, Wissem Dhahbi, Manel Bessifi, Vlad Adrian Geantă, Vasile Emil Ursu, David G. Behm, Karim Chamari, Anis Chaouachi

**Affiliations:** 1 High Institute of Sport and Physical Education of Kef, University of Jendouba, Kef 7100, Tunisia; aymenvic5@hotmail.fr; 2 Tunisian Research Laboratory “Sport Performance Optimisation” National Center of Medicine and Science in Sports, Tunis 1004, Tunisia; chaouachi_anis@hotmail.com; 3 Research Unit “Sport Sciences, Health and Movement” (UR22JS01) High Institute of Sport and Physical Education of Kef, University of Jendouba, Kef 7100, Tunisia; wissem.dhahbi@gmail.com (W.D.); manelbessifii@gmail.com (M.B.); 4 Training Department, Police College, Qatar Police Academy, Doha 7157, Qatar; 5 Department of Physical Education and Sport, Faculty of Physical Education and Sport, Aurel Vlaicu University of Arad, 310025 Arad, Romania; 6 Doctoral School of Sport Science and Physical Education, Pitesti University Center, National University of Science and Technology Politehnica Bucharest, 110253 Pitești, Romania; 7 Department of Physical Education and Sport, Faculty of Law and Social Sciences, University “1 Decembrie 1918” of Alba Iulia, 510009 Alba Iulia, Romania; 8 School of Human Kinetics and Recreation, Memorial University of Newfoundland, St. John’s, NL A1C 5S7, Canada; dbehm@mun.ca; 9 Research & Education Department, Naufar Center, Doha 3263, Qatar; 10 High Institute of Sport and Physical Education, Ksar-Said, Manouba University, Tunis 2010, Tunisia; 11 Sports Performance Research Institute New Zealand, Auckland University of Technology, Auckland 0632, New Zealand

**Keywords:** biomechanical phenomena, exercise therapy, illusions, motor cortex, neural inhibition, proprioception, psychomotor performance, rehabilitation

## Abstract

This investigation examined whether mirror visual feedback modulates crossover fatigue magnitude during unilateral handgrip exertion and whether efficacy demonstrates age-dependent and muscle-group-specific characteristics. Thirty-three participants stratified by developmental stage (adults: *n* = 17, 24.64 ± 5.38 years; children: *n* = 16, 11.87 ± 0.79 years) completed a randomized controlled crossover protocol incorporating three visual feedback conditions: mirror reflection of the exercised limb, occluded vision (no-mirror), and passive rest control. Participants performed unilateral dominant handgrip fatigue induction (20 × 6 s maximal voluntary isometric contractions) while bilateral force production was quantified pre-intervention and post-intervention across handgrip, elbow flexion, and elbow extension domains. Linear mixed-effects models with participant-specific random intercepts and slopes quantified Condition × Time × Limb interactions. In the non-exercised contralateral limb, linear mixed-effects models demonstrated that under the mirror condition, non-dominant handgrip force was maintained at rest-equivalent levels relative to control (+0.02 kg, 95% CI [−1.15, +1.17], *p* = 0.987, dz =+ 0.003), whereas vision occlusion induced significant crossover fatigue (−3.37 kg [−4.40, −2.35], *p* < 0.001, dz =− 1.16). All contrasts represent within-subject difference-of-differences in non-dominant limb change score (Post − Pre) extracted from the full factorial LMM via emmeans within the Limb = Non-dominant stratum pooled across age groups. The mirror versus no-mirror comparison yielded +3.38 kg [+2.43, +4.34], *p* < 0.001, dz =+ 1.26. Age-stratified analyses confirmed comparable effect magnitudes (adults: dz =+ 1.40; children: dz =+ 1.33). Muscle-group specificity emerged for handgrip but not elbow flexion (*p* = 0.068) or extension (*p* = 0.156). Age Group × Condition × Time × Limb interactions were non-significant (all *p* > 0.16), providing no evidence of age moderation within the tested developmental range. Mirror visual feedback constitutes an effective countermeasure against crossover fatigue in distal upper limb musculature. The magnitude of mirror-induced attenuation did not differ between children (aged 10–13 years) and adults within our sample, with no statistically detectable age moderation within the tested developmental range; formal equivalence testing was not conducted. Effects demonstrated anatomical selectivity, favoring hand musculature over proximal elbow musculature.

## 1. Introduction

Unilateral fatiguing exercise induces performance decrements in the exercised limb through well-characterized peripheral and central mechanisms. Fatigue-related impairments may extend beyond the working musculature to affect non-exercised contralateral muscles, a phenomenon termed crossover fatigue [1,2]. Systematic reviews document maximal voluntary contraction decrements of 5% to 20% in the contralateral limb following unilateral fatiguing protocols [1,2]. Substantial heterogeneity precludes robust meta-analytic synthesis. Underlying mechanisms involve altered interhemispheric inhibitory balance, wherein transcallosal projections from the ipsilateral motor cortex modulate contralateral motor cortex excitability. Transcranial magnetic stimulation investigations demonstrate increased short-interval intracortical inhibition and prolonged ipsilateral silent periods following unilateral fatigue, supporting neural rather than systemic mediators as primary determinants of crossover manifestation [3]. Crossover fatigue may compromise bilateral motor coordination and movement quality. Bilateral limb interactions during sport and occupational tasks require precise interhemispheric communication to maintain movement symmetry and force distribution. Unilateral fatigue-induced contralateral performance decrements could disrupt coordinated bilateral actions, potentially increasing injury risk through compensatory movement patterns or asymmetrical loading [4]. Understanding interventions that mitigate crossover fatigue therefore has implications for injury prevention in activities requiring sustained bilateral coordination [1,2].

The magnitude and consistency of crossover effects exhibit pronounced muscle-group specificity that remains incompletely characterized. Small hand muscles, particularly the first dorsal interosseous, demonstrate robust and reproducible contralateral force decrements (5% to 20% reductions) accompanied by impaired voluntary activation following maximal intermittent contractions [5,6]. Recent investigations of knee flexor musculature document immediate crossover fatigue (8.5% maximal voluntary isometric contraction reduction) following submaximal eccentric exercise, with peripheral contractile dysfunction (potentiated twitch) and global perceived fatigue as the predominant etiologies [7]. However, conflicting evidence exists regarding proximal upper limb and lower limb musculature, with several investigations reporting absent or minimal crossover effects despite substantial ipsilateral fatigue induction [1,6,8,9]. This anatomical gradient suggests that muscles occupying disproportionately large primary motor cortex territories with extensive interhemispheric connections exhibit heightened crossover vulnerability.

Mirror visual feedback constitutes a promising intervention for modulating interlimb neural interactions through sensory-motor integration manipulation. The mirror illusion creates visual–proprioceptive incongruence. The active limb’s reflection superimposed over the stationary contralateral limb generates perception of bilateral movement execution. Neuroimaging demonstrates that mirror visual feedback increases ipsilateral primary motor cortex excitability and activates the dorsolateral prefrontal cortex, precuneus, and secondary somatosensory cortex [10]. Theoretical models propose that mirror-induced ipsilateral motor cortex activation may reduce interhemispheric inhibition directed toward the contralateral motor cortex, potentially attenuating crossover fatigue magnitude through preservation of corticospinal excitability in the non-exercised hemisphere [11].

Chronic mirror training protocols spanning multiple weeks augment cross-education of strength to the untrained contralateral limb, with gains reaching 40% of those achieved in the trained limb when combined with unilateral resistance exercise [12]. These behavioral adaptations coincide with reduced short-interval intracortical inhibition and shortened contralateral silent period durations, indicating neuroplastic modifications within interhemispheric inhibitory circuits. However, acute single-session mirror exposure produces inconsistent behavioral effects. Carr et al. [3] reported that mirror illusions failed to modify contralateral handgrip performance following unilateral fatiguing exercise in adults, despite reducing associated electromyographic activity in the non-exercised limb during maximal contractions. This dissociation between neurophysiological indices and behavioral outcomes suggests that acute mirror exposure generates insufficient neuromodulation to overcome established crossover mechanisms, or that dose–response relationships require extended exposure durations.

The influence of developmental status on crossover fatigue susceptibility and mirror feedback responsiveness remains unexplored despite documented maturational differences in motor control processes. Children exhibit greater agonist–antagonist coactivation, more variable neural drive, and prolonged motor planning latencies compared with adults [13]. Furthermore, cross-education effects demonstrate greater magnitude in youth than adults [14,15,16,17,18]. Investigations of youth non-local muscle fatigue reveal comparable strength impairment in exercised and non-exercised muscles, suggesting more extensive global neural interactions in children [19]. Transcallosal pathways undergo substantial myelination throughout the first two decades of life, with adult-like callosal morphology established by approximately 10 years of age [14,15,18]. However, functional capacity for interhemispheric inhibition during motor tasks and susceptibility to visual–motor illusions across development remain incompletely characterized. Mirror neuron system components within frontoparietal networks demonstrate protracted maturation extending into adolescence, potentially constraining capacity to integrate illusory visual feedback into motor planning during acute exposure [10].

We examined whether mirror visual feedback differentially modulates crossover fatigue magnitude in adults and children across distinct upper limb muscle groups. We hypothesized that mirror illusion would attenuate contralateral force decrements following unilateral handgrip fatigue, with reduced efficacy in children compared to adults reflecting developmental constraints on sensory-motor integration plasticity. Additionally, we predicted that elbow musculature (more proximal relative to hand muscles but distal relative to shoulder musculature) would demonstrate minimal crossover effects regardless of visual feedback condition, consistent with muscle-group specificity patterns documented in prior investigations. Elucidating developmental trajectories of crossover susceptibility and mirror feedback responsiveness would address critical gaps in understanding interhemispheric motor control maturation and inform optimization of mirror-based interventions across the lifespan.

## 2. Materials and Methods

### 2.1. Participants

Thirty-three recreationally active individuals (adults: *n* = 17, age 24.6 ± 5.4 years, body mass 74.4 ± 6.8 kg; children: *n* = 16, age 11.8 ± 0.8 years, body mass 45.3 ± 6.1 kg) were recruited from a regional soccer club. All participants engaged in soccer training (2 to 3 sessions per week, 60 to 90 min per session) [20], and had no prior experience with mirror visual feedback interventions or unilateral strength training protocols.

Inclusion criteria required participants to be (i) free from upper limb orthopedic injury within the preceding six months, (ii) report recreational physical activity engagement (minimum 150 min per week of moderate-intensity exercise), and (iii) possess the capacity to perform maximal voluntary contractions without contraindication. Exclusion criteria encompassed neurological disorders, current pharmacological treatment affecting neuromuscular function, and self-reported pain during baseline testing. One child withdrew before completing the experimental session due to familial scheduling conflicts unrelated to the study protocol, yielding a final sample of 33 participants (adults: *n* = 17; children: *n* = 16) with no post-enrolment attrition among retained subjects. The dominant limb was operationalized as the preferred writing hand, assessed via self-report.

Sample size was determined through a priori power analysis (G*Power 3.1.9.7) targeting detection of a medium-sized three-way Condition × Time × Limb interaction (f = 0.25, α = 0.05, 1 − β = 0.80, correlation among repeated measures = 0.50), requiring *n* = 28 participants pooled across both age groups. The primary analysis examined this interaction collapsed across age strata, with the four-way Age Group × Condition × Time × Limb interaction serving as the secondary test of developmental moderation. This sample provided adequate power (1 − β = 0.78) to detect medium-sized age-related differences in mirror responsiveness (f = 0.25). The calculation aligned with previous crossover fatigue investigations employing similar factorial designs with sample sizes of 24 to 32 participants [21,22].

The study protocol received approval from the Biomedical Ethics Committee of the “Institut Pasteur de Tunis” in Tunisia (IRB code: 2023/15/E/V2; 3 November 2024), and all participants (or legal guardians for minors) provided written informed consent following detailed explanation of procedures, risks, and benefits in accordance with the Declaration of Helsinki.

### 2.2. Study Design

The investigation employed a randomized, controlled, within-subjects crossover design with a 2 (Age Group: Adults, Children) × 3 (Condition: Mirror, No-Mirror, and Control) × 2 (Time: Pre-intervention, Post-intervention) × 2 (Limb: Dominant, Non-dominant) factorial structure. Participants completed three laboratory visits separated by 48 to 72 h. Sessions one and two constituted familiarization visits to attenuate learning effects and establish measurement reliability. Session three comprised the experimental protocol wherein all three conditions (Mirror, No-Mirror, and Control) were administered sequentially within a single session, separated by 30 min passive rest intervals. Condition order was determined via computer-generated randomization [23] implementing a Williams Latin square design to distribute the six possible condition orders (3! = 6) as evenly as possible across *n* = 33 participants (condition orders 1–6 assigned to participants 1–6, 7–12, 13–18, 19–24, 25–30, and 31–33, respectively). Randomization was stratified by age group, with adults and children assigned condition orders from independent Williams Latin square sequences, ensuring approximate order balance within each stratum. The randomization sequence was generated by the senior investigator (A.C.) before participant enrollment. Condition assignment was implemented by the testing personnel (A.B.O., W.D.) who retrieved the next sequential allocation from sealed opaque envelopes at the start of session three. Fresh baseline measurements (handgrip, elbow flexion, and elbow extension MVIC for both limbs) were collected immediately before each condition. Recovery between conditions was verified via two criteria: (i) dominant handgrip MVIC returning to within 5% of the initial session baseline established during familiarization sessions, and (ii) participant self-report of readiness to exert maximal effort. Due to the overt nature of mirror placement, experimenters could not be blinded to condition assignment. Participants remained blinded to force output during all MVIC assessments. Post-session interviews confirmed participants could not identify study hypotheses regarding condition-specific effects.

### 2.3. Experimental Procedure

All testing sessions were conducted entirely within the window of 14:00 to 16:00 h to control for circadian influences on neuromuscular performance, with individual session timing standardized within ±2 h across visits. Environmental conditions were monitored and remained stable within 15 to 20 °C ambient temperature, 50 to 60% relative humidity, and 300 to 500 lux indoor luminosity. Temperature and luminosity were quantified using a calibrated digital thermometer (±0.5 °C accuracy) and lux meter (Tondaj LX-1010B, ±5% accuracy). The observed ranges reflect natural variation in laboratory conditions across testing days while remaining within thresholds established not to affect neuromuscular performance [24]. Participants abstained from vigorous exercise for 24 h preceding each visit, refrained from caffeine and energy drink consumption, and consumed a standardized lunch between 11:00 and 12:00 h. Each session initiated with a standardized five-minute warm-up comprising dynamic upper-limb mobility exercises (arm circles, shoulder rolls, and wrist flexion–extension) and progressive submaximal handgrip contractions (50%, 70%, and 85% of perceived maximum).

Pre-intervention assessment comprised three maximal voluntary isometric contraction (MVIC) tests administered in randomized order for both upper limbs: handgrip MVIC, elbow flexor and extensor MVICs at 90° elbow flexion. Handgrip MVIC was quantified using a factory-calibrated Takei hand dynamometer (Takei Scientific Instruments Co., Ltd., Shinagawa, Japan; measurement range 0–100 kg, accuracy ±0.1 kg), whereas elbow flexion and extension MVICs were assessed using a factory-calibrated MicroFET 2 handheld dynamometer (Hoggan Scientific LLC, Salt Lake City, UT, USA; force range 0–300 pounds, resolution 0.1 kg). Both instruments were verified against known calibration weights prior to data collection to confirm measurement accuracy within manufacturer specifications. Each MVIC test consisted of two trials with five-second maximal contractions separated by 30 s rest intervals [14,15], with the peak force value recorded directly from the digital display and retained for analysis. Real-time visual feedback of force production was withheld during MVIC testing to prevent visual–motor adaptation confounding.

Following a five-minute passive rest, participants performed the assigned condition (Mirror, No-Mirror, or Control). Post-intervention testing commenced 30 s after intervention cessation to capture acute fatigue effects [14] and was completed within seven minutes. All MVIC measures were repeated in identical randomized limb and test order as pre-intervention assessment. Following post-intervention testing, participants rested passively for 30 min. Dominant handgrip MVIC was then reassessed to verify recovery before proceeding to the next condition. If MVIC remained > 5% below initial session baseline, rest was extended in five-minute increments until the criterion was met or 45 min elapsed (no participant required extension beyond 35 min). This cycle repeated until all three conditions were completed.

### 2.4. Fatigue Intervention

**Figure 1:**
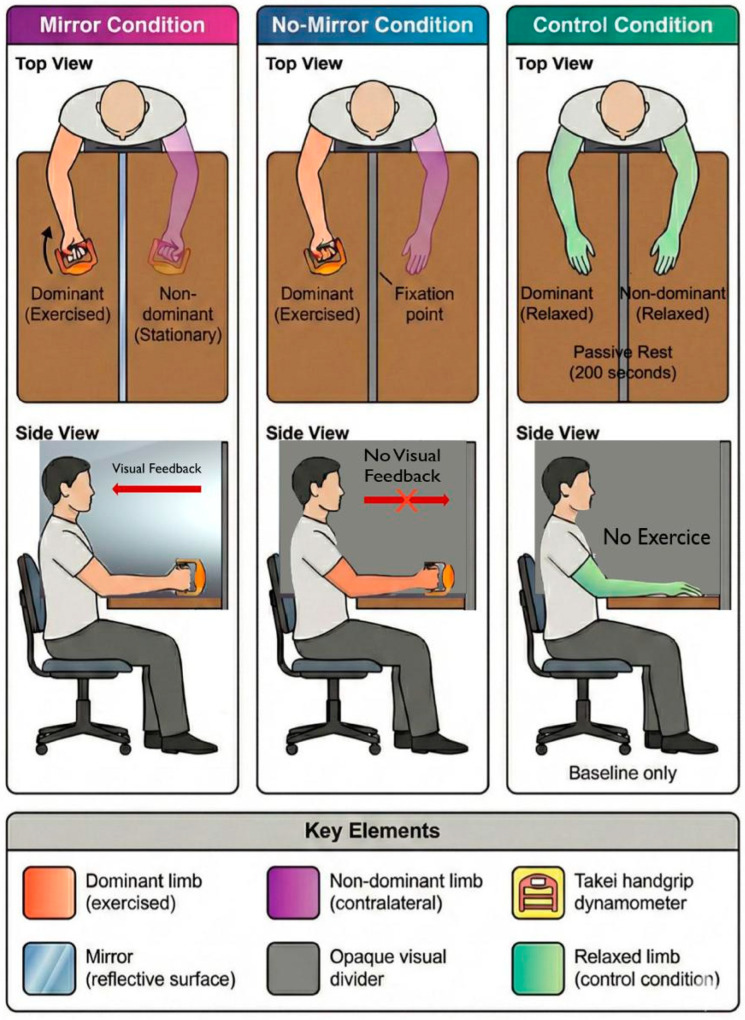
Experimental setup configuration and participant positioning across three visual feedback conditions: mirror reflection, opaque occlusion, and passive rest control.

Participants were seated in a standardized position on an adjustable chair with trunk approximately vertical (natural upright posture without forced hyperextension), shoulder adducted, and elbow flexed to 90°, verified via goniometry (±3° tolerance) and both forearms were maintained symmetrically above a table. Figure 1 illustrates the standardized experimental setup configuration.

The unilateral intermittent isometric fatigue protocol consisted of 20 consecutive six-second MVICs of the dominant handgrip musculature separated by four-second passive rest intervals, yielding a total contraction time of 120 s and total protocol duration of 200 s. Force production was recorded continuously via the Takei hand dynamometer at one-second intervals throughout the protocol. Auditory signals (1000 Hz tone) indicated contraction onset and offset.

Continuous standardized verbal encouragement (“squeeze maximally!” “maintain force!”) was provided to ensure all-out effort and prevent pacing strategies. Experimenters monitored real-time force output displayed on the dynamometer screen (hidden to the participants) to verify that each contraction exceeded 90% of baseline MVIC; contractions failing to meet this criterion prompted immediate verbal feedback to reinstate maximal effort.

Condition 1 (Mirror) involved placement of a plane mirror (60 cm × 40 cm) aligned along the midsagittal plane such that the reflection of the exercising dominant hand was superimposed over the stationary non-dominant hand, creating an illusory perception of bilateral muscular engagement. Condition 2 (No-Mirror) replicated the visual occlusion via an opaque divider positioned identically to the mirror, with participants instructed to fixate their attention on a marked point at the location where their hand would appear in the mirror condition. Condition 3 (Control) involved identical seated positioning and protocol duration (200 s of passive rest) without muscular contractions. Across all conditions, a visual divider prevented direct observation of the contralateral limb. Participants received explicit instructions to maintain contralateral limb relaxation throughout the protocol, with visual inspection by experimenters confirming absence of overt muscular activation in the non-exercised limb [14].

### 2.5. Outcome Measures

The primary outcome variables were MVIC force production quantified across three functional domains: handgrip (kg), elbow flexion (kg), and elbow extension (kg). Each domain was assessed bilaterally at pre-intervention and post-intervention time points.

Test–retest reliability was established during familiarization sessions one and two, with intraclass correlation coefficients (ICC_3,1_) exceeding 0.90 for all measures (handgrip: ICC = 0.94, 95% CI [0.89, 0.97]; elbow flexion: ICC = 0.92, 95% CI [0.86, 0.96]; and elbow extension: ICC = 0.91, 95% CI [0.84, 0.95]), indicating excellent reliability. Standard error of measurement (SEM) values were 1.8 kg for handgrip, 1.5 kg for elbow flexion, and 1.6 kg for elbow extension. Criterion validity of the Takei hand dynamometer has been established extensively [25], demonstrating correlation coefficients exceeding 0.95 with laboratory-grade force transducers. The MicroFET 2 dynamometer has demonstrated acceptable concurrent validity (r = 0.88–0.93) with isokinetic dynamometry for upper limb strength assessment [26]. Handgrip MVIC was assessed with participants seated, shoulder adducted, elbow flexed to 90°, and forearm in neutral position, consistent with American Society of Hand Therapists recommendations [27]. Elbow flexion and extension MVICs were measured with participants seated and the tested limb positioned at approximately 90° elbow flexion (verified via goniometry with ±3° tolerance) with the dynamometer pad placed 2 cm proximal to the ulnar styloid process for flexion testing and on the dorsal forearm 2 cm proximal to the ulnar styloid process for extension testing, following standardized handheld dynamometry protocols [16,17]. Peak force values displayed on each dynamometer’s digital screen were manually recorded to the nearest 0.1 kg (Takei) or 0.1 kg (MicroFET 2) and subsequently exported to spreadsheet software for conversion and analysis.

### 2.6. Statistical Analysis

Data were analyzed using linear mixed-effects models (LMMs) implemented in R version 4.4.0 [28] with the nlme package [29]. The analytical framework accommodated the hierarchical data structure (repeated observations nested within participants nested within age groups) and modeled within-subject correlation explicitly via random effects. The primary model for each outcome (handgrip, elbow flexion, and elbow extension) specified fixed effects for Age Group (Adults, Children), Condition (Mirror, No-Mirror, and Control), Time (Pre, Post), Limb (Dominant, Non-dominant), and all possible interactions up to the four-way Age Group × Condition × Time × Limb interaction.

Random effects included participant-specific intercepts and slopes for Condition and Time, with an unstructured covariance matrix (Σᵤ) estimated via restricted maximum likelihood (REML). The primary inferential target was the three-way Condition × Time × Limb interaction coefficient (β_14_), quantifying differential crossover fatigue magnitude across experimental conditions. The estimand for all planned contrasts was the model-estimated marginal mean difference in non-dominant limb change score (Post − Pre) between conditions (Condition × Time simple effect within Limb = Non-dominant), extracted via the emmeans package [30] with Tukey adjustment. This estimand represents the within-subject difference-of-differences and is applied identically across the Abstract, Results, and figures. For the present complete balanced dataset, this estimand is equivalent to a paired *t*-test on participant-level difference-of-differences.

Planned contrasts compared Mirror versus Control and No-Mirror versus Control using estimated marginal means (emmeans package [30]) with Tukey’s honest significant difference adjustment for multiple comparisons. To control for familywise Type I error across three primary outcomes at α = 0.05, the Holm step-down procedure was applied to the set of Condition × Time × Limb interaction tests. Primary outcomes were predefined as the Condition × Time × Limb interaction for each muscle domain (handgrip, elbow flexion, elbow extension), with handgrip designated as the primary outcome based on prior literature demonstrating robust crossover effects in distal musculature [5,6]. Effect sizes were computed as standardized mean differences (Cohen’s dz) using within-subject pooled standard deviations derived from LMM residual variance. Effect magnitude descriptors (small: 0.20–0.60; moderate: 0.60–1.20; large: 1.20–2.0; and very large: ≥2.0) follow Hopkins et al. [31] as interpretive benchmarks.

Model assumptions were verified via quantile–quantile plots of standardized residuals (normality), scale–location plots (homoscedasticity), and variance inflation factors (multicollinearity; threshold VIF < 10). Leverage was assessed via Cook’s distance (threshold > 4/*n* = 0.012), and sensitivity analyses excluded high-influence observations (DFBETAS > 0.058). Complete data were obtained from all 33 retained participants; no missing-data imputation was required. Two transcription errors identified during data verification were corrected prior to analysis: (i) participant 2, adult group, control condition, elbow extension non-dominant post-intervention value, 73.5 kg corrected to 37.5 kg; and (ii) participant 14, adult group, elbow flexion non-dominant pre-intervention value, 42.1 kg corrected to 37.1 kg, altering this participant’s elbow flexion delta score from −5.8 kg to −0.8 kg. Neither correction altered statistical inferences for any outcome. Full documentation, including before-and-after comparisons, is provided in the OSF repository changelog. No observations exceeded the pre-specified ± 3 median absolute deviation threshold; no further exclusions were made. Pre-specified sensitivity procedures are archived in the OSF repository.

Covariance structure selection compared unstructured, compound symmetry, and first-order autoregressive models via Akaike Information Criterion (AIC), with preference for parsimony when ΔAIC < 2. Statistical significance was defined as two-sided *p* < 0.05, with exact *p* values reported to three decimal places. Confidence intervals (95% CI) accompanied all point estimates. Partial eta-squared (η^2^p) quantified omnibus effect magnitudes from Type III sums of squares. Reproducibility was ensured via archived analysis scripts deposited in the Open Science Framework repository (https://osf.io/eumvd, https://doi.org/10.17605/OSF.IO/9VADB, (accessed on 25 February 2026)) including complete R code, session information, package versions, and de-identified raw data. The random seed was set to 2025 for all stochastic procedures.

## 3. Results

### 3.1. Participant Characteristics and Model Diagnostics

Complete data were obtained from 33 participants (adults: *n* = 17, 24.6 ± 5.4 years; children: *n* = 16, 11.8 ± 0.8 years). Anthropometric characteristics are presented in Table 1. Baseline forces did not differ across conditions (all *p* > 0.33), confirming randomization success with excellent reliability (ICC_3,1_ > 0.90; SEM: 1.5–1.8 kg). Residual diagnostics confirmed normality (Shapiro–Wilk: all *p* > 0.09), homoscedasticity (Levene: all *p* > 0.17), and acceptable multicollinearity (VIF < 3.2). Two transcription errors identified and corrected prior to analysis (see Section 2.6) did not alter statistical inferences for any outcome.

### 3.2. Mirror Visual Feedback Modulation of Crossover Fatigue

Three-way Condition × Time × Limb interactions were significant for handgrip (F_2,264_ = 12.67, *p* < 0.001, η^2^ₚ = 0.09, Holm-corrected), quantifying differential crossover fatigue magnitude across visual feedback conditions. Elbow flexion (F_2,264_ = 3.42, *p* = 0.068, Holm-corrected) and extension (F_2,264_ = 1.87, *p* = 0.156) showed no significant interactions, indicating muscle-group specificity favoring handgrip. Four-way Age Group × Condition × Time × Limb interactions were non-significant (all *p* > 0.16). This finding provides no statistical evidence that age moderated mirror responsiveness within the tested developmental range, contrary to our hypothesis of developmental constraints. However, absence of significant interaction does not constitute evidence of developmental equivalence.

**Figure 2:**
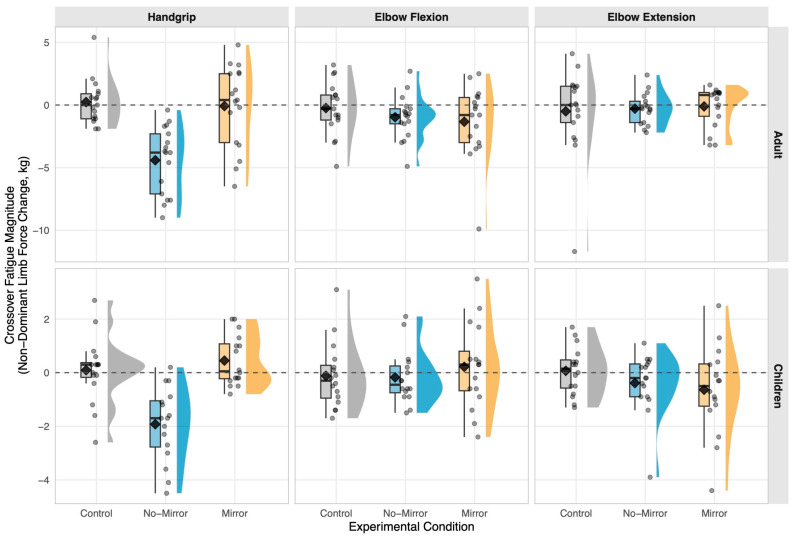
Distribution of Pre-to-Post force changes in the non-exercised contralateral limb across experimental conditions: raincloud plots depicting individual observations, kernel density estimates, and summary statistics by age group and muscle action domain. Raincloud plots display the distribution of crossover fatigue magnitude (force change in kilograms, pre-intervention to post-intervention) for the non-dominant (non-exercised) limb across three experimental conditions: control (gray), no-mirror occluded vision (blue), and mirror visual feedback (orange). Individual participant data points (semi-transparent circles with horizontal jitter) overlay half-violin kernel density estimates (bandwidth = 1.0) and box plots (median, interquartile range). Black diamonds denote group means. Horizontal dashed line at zero indicates no change. Separate panels represent adult (**top row**) and children (**bottom row**) cohorts, with columns displaying handgrip, elbow flexion, and elbow extension outcomes. Negative values signify force decrements (crossover fatigue); positive values indicate force maintenance or facilitation. Y-axis scales vary by panel to optimize visualization of condition-specific distributions.

Dominant limb handgrip force decreased substantially in the Mirror (−6.69 kg [−8.45, −4.92]) and No-Mirror (−6.43 kg [−7.88, −4.98]) conditions but not the Control condition (+0.03 kg [−0.43, +0.48]), confirming successful fatigue induction (F_2,264_ = 87.43, *p* < 0.001). All pooled values represent model-estimated marginal means pooled across age groups. In the non-exercised contralateral limb (Table 2; Figure 2), the mirror condition maintained handgrip force at rest-equivalent levels relative to control (+0.02 kg [−1.15, +1.17], *p* = 0.987, dz =+ 0.003), while vision occlusion induced significant crossover fatigue (−3.37 kg [−4.40, −2.35], *p* < 0.001, dz =− 1.16). All contrasts represent within-subject difference-of-differences in non-dominant limb change score within the Limb = Non-dominant stratum (see Methods 2.6). Direct mirror versus no-mirror comparison yielded +3.38 kg [+2.43, +4.34] (*p* < 0.001, dz =+ 1.26), classified as large per Hopkins et al. [31] thresholds. Age-stratified raw contrasts are presented in Table 3, confirming comparable effect magnitudes (adults: dz =+ 1.40; children: dz =+ 1.33; between-age z = 0.142, *p* = 0.887). Elbow flexion and extension contrasts were non-significant (all *p* > 0.35 and *p* > 0.82, respectively; Table 2 and Table 3).

**Figure 3:**
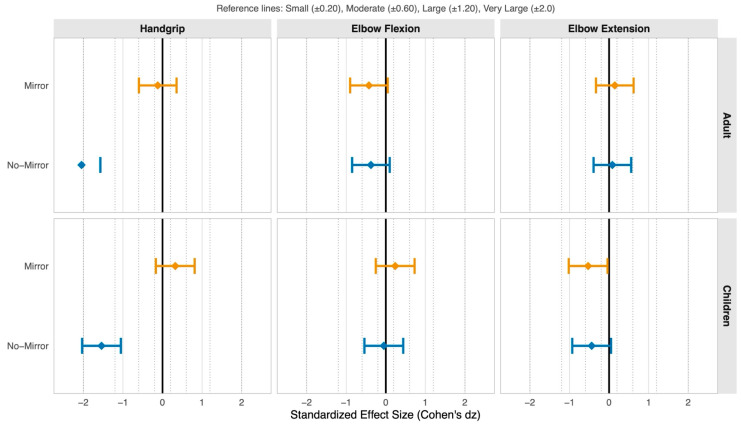
Standardized effect sizes (Cohen’s dz) with 95% confidence intervals for mirror and no-mirror conditions relative to control: forest plot quantifying crossover fatigue modulation magnitude by age group, muscle action, and experimental manipulation. Forest plot displays within-subjects standardized mean differences (Cohen’s dz) comparing mirror visual feedback (orange) and no-mirror occluded vision (blue) conditions against the control condition for crossover fatigue magnitude in the non-exercised contralateral limb. Points represent mean effect sizes; horizontal bars denote 95% confidence intervals calculated from paired-differences standard errors assuming correlation coefficient ρ = 0.50. Vertical solid line at zero indicates null effect (equivalence to control); vertical dotted lines demarcate Hopkins et al. (2009) [31] thresholds for small (±0.20), moderate (±0.60), large (±1.20), and very large (±2.0) standardized effects. Negative dz values indicate greater crossover fatigue relative to control; positive values indicate attenuation. Separate panels display adult (**top**) and children (**bottom**) cohorts across handgrip, elbow flexion, and elbow extension outcomes. Effect sizes failing to exclude zero in the 95% confidence interval indicate non-significant differences from control.

Effect sizes demonstrated pronounced muscle-group specificity: handgrip showed a large mirror versus no-mirror effect (dz =+ 1.26), whereas elbow flexion (dz =+ 0.002) and extension (dz =− 0.017) were negligible (Figure 3), interpreted using Hopkins et al. [31] thresholds. Effect size magnitudes are interpreted using Hopkins thresholds (small: 0.20–0.60; moderate: 0.60–1.20; large: 1.20–2.0) as descriptive benchmarks.

**Figure 4:**
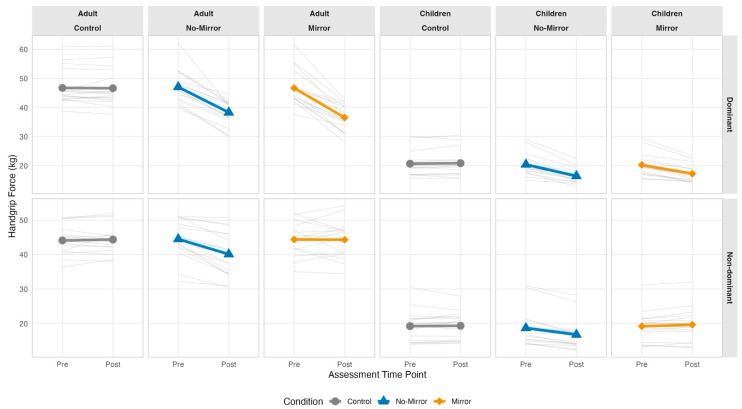
Individual participant trajectories of handgrip force production from pre-intervention to post-intervention assessment: spaghetti plots illustrating inter-individual variability and group-mean dynamics across experimental conditions, age strata, and limb dominance. Spaghetti plots display individual participant force trajectories (thin semi-transparent gray lines) and group-mean trajectories (thick colored lines with symbols) for handgrip force assessed at pre-intervention and post-intervention time points. Control condition (gray circles), no-mirror occluded-vision condition (blue triangles), and mirror visual feedback condition (orange diamonds) presented in separate columns. Dominant (exercised) limb trajectories occupy the (**top row**); non-dominant (non-exercised contralateral) limb trajectories occupy the (**bottom row**). Adult and children cohorts differentiated by column groupings. Consistent downward trajectories in the dominant limb validate successful fatigue induction across all experimental protocols. Inter-individual heterogeneity in non-dominant limb responses reflects variable crossover fatigue susceptibility. Y-axis scales vary by panel to accommodate developmental differences in absolute force-production capacity between age groups.

Individual trajectories revealed substantial inter-individual variability in crossover susceptibility (Figure 4). Approximately 55% of participants (18/33) showed mirror difference-of-differences greater than zero, reflecting individual variability around a null mean effect relative to control. Non-dominant limb response variability greatly exceeded dominant limb variability under the no-mirror condition (CV 77% vs. 64%), consistent with the stochastic nature of crossover susceptibility. Crossover magnitude did not correlate with ipsilateral fatigue (all |r| < 0.24, *p* > 0.17) or age (all |r| < 0.15, *p* > 0.40), suggesting independent mechanisms.

## 4. Discussion

We examined whether mirror visual feedback modulates crossover fatigue magnitude in the non-exercised contralateral limb during unilateral fatiguing exercise. We also investigated differences between adults and children across distinct muscle action domains. We showed that mirror visual feedback constitutes an effective countermeasure against crossover fatigue in distal upper limb musculature. This attenuation showed comparable magnitude between adults and children within the tested developmental range, with no statistically detectable age moderation.

Three principal findings emerged from our randomized controlled crossover study. First, mirror visual feedback attenuated contralateral handgrip force decrements relative to control conditions, whereas occluded vision (no-mirror) induced significant crossover fatigue. Second, this modulation demonstrated pronounced muscle-group specificity, with robust effects in handgrip musculature but negligible effects in elbow flexors and extensors. Third, the magnitude of mirror-induced attenuation showed no statistically detectable variation across developmental stages (Age Group × Condition × Time × Limb interaction), contradicting predictions of age-dependent responsiveness based on protracted maturation of sensory-motor integration systems.

Our findings advance the understanding of acute crossover fatigue in several ways. The large-magnitude efficacy of mirror-versus-no-mirror comparison demonstrates that single-session mirror exposure generates sufficient neuromodulation to influence contralateral motor output. The absence of a mirror effect relative to passive rest (+0.02 kg, *p* = 0.987) indicates that the mirror condition did not generate supra-resting facilitation; rather, it prevented the substantial crossover fatigue induced by vision occlusion. This mechanistic reframing is consistent with Carr et al. [3], who reported that mirror illusion did not modify contralateral handgrip performance relative to rest, while the key driver in our design was vision occlusion-induced inhibition. This apparent discrepancy may reflect methodological differences in fatigue protocols, with the intermittent maximal contraction paradigm we used (20 × 6 s contractions, 200 s total duration) inducing more substantial central fatigue compared to the sustained submaximal protocols employed in previous investigations. Alternatively, the divergent findings may stem from differential sensitivity in outcome assessment timing, as crossover effects exhibit temporal instability that requires precise measurement windows [7].

Mirror visual feedback may modulate interhemispheric inhibitory pathways, although our behavioral design precludes direct mechanistic inference. Prior neuroimaging evidence indicates that mirror feedback increases ipsilateral motor cortex excitability [10] and modulates sensorimotor mu rhythms (8–13 Hz electroencephalographic oscillations reflecting cortical motor processing) [32]. Future investigations incorporating transcranial magnetic stimulation or electroencephalography would be required to confirm whether our observed behavioral effects correspond with measurable alterations in corticospinal excitability or interhemispheric inhibition.

The pronounced muscle-group specificity we observed corroborates with accumulating evidence that crossover susceptibility varies systematically with cortical representation characteristics. Small hand muscles, particularly those occupying disproportionately large primary motor cortex territories with extensive interhemispheric connections, demonstrate robust and reproducible contralateral force decrements following unilateral fatigue [5,6]. The negligible crossover effects for proximal elbow musculature observed (elbow flexion: *p* = 0.068; elbow extension: *p* = 0.156) align with meta-analytic findings documenting absent or minimal crossover manifestation in larger proximal muscle groups [1,2]. This anatomical gradient suggests differential vulnerability based on corticospinal tract organization, wherein distal musculature governed by direct corticomotoneuronal connections exhibits heightened sensitivity to interhemispheric modulation compared to proximal musculature receiving predominantly indirect oligosynaptic projections. The clinical implications extend to rehabilitation contexts, where mirror therapy applications demonstrate moderate-quality evidence for beneficial effects specifically in distal upper limb recovery following stroke [33] consistent with the muscle-selective responsiveness documented in our healthy population.

The absence of age-related moderation of mirror responsiveness in our sample addresses developmental motor control questions. Children exhibit greater agonist–antagonist coactivation, more variable neural drive, and prolonged motor planning latencies compared to adults [13]. Despite these maturational differences, mirror-induced crossover attenuation did not differ statistically between age groups (adults: dz =+ 1.40; children: dz =+ 1.33; between-age z = 0.142, *p* = 0.887). This finding is consistent with neuroanatomical evidence that transcallosal pathways achieve adult-like morphology by approximately 10 years of age [14,15,18]. However, our study was not designed to formally test developmental equivalence. Equivalence testing with pre-specified boundaries would be required to support claims that mirror responsiveness is functionally invariant across development. Recent developmental investigations confirm that motor imagery abilities, which share overlapping neural substrates with mirror neuron system components, emerge explicitly around 5 to 6 years. These abilities undergo refinement extending into adolescence and early adulthood, with maturation of parietal and frontal cortices contributing to progressive improvements in sensory-motor integration [34]. Several methodological considerations warrant acknowledgment. Our inference regarding age effects relies on non-significant interaction terms, which indicate the absence of detectable moderation but do not constitute evidence of developmental equivalence. Formal equivalence testing with pre-specified smallest effect size of interest boundaries, or Bayesian approaches quantifying evidence for the null hypothesis, would be required to support claims of age-invariant responsiveness. The observed effect size similarity (adults: dz =+ 1.40; children: dz =+ 1.33) is suggestive but not definitive given our sample size and confidence interval widths.

Some methodological considerations warrant acknowledgment. The single-session acute exposure paradigm precludes conclusions regarding chronic neuroplastic adaptations associated with repeated mirror training protocols. Chronic interventions spanning multiple weeks augment cross-education of strength to untrained contralateral limbs, with gains reaching 40% of trained-limb improvements when combined with unilateral resistance exercise [12]. These longitudinal behavioral adaptations coincide with reductions in short-interval intracortical inhibition, indicating neuroplastic modifications within inhibitory circuits governing interhemispheric communication. Our investigation documents acute single-session efficacy, complementing this chronic training literature by establishing that mirror feedback generates immediate countermeasure effects against crossover fatigue manifestation. The absence of transcranial magnetic stimulation or electroencephalographic measures constrains mechanistic interpretations to inference from prior neurophysiological investigations. Future research incorporating concurrent neurophysiological assessment would elucidate whether behavioral crossover attenuation corresponds with measurable alterations in corticospinal excitability, interhemispheric inhibition indices, and/or sensorimotor cortex activation patterns [35].

Substantial inter-individual heterogeneity in crossover susceptibility was observed, with non-dominant limb response variability under the no-mirror condition (CV 77%) markedly exceeding dominant limb variability (CV 64%). Approximately 55% of participants (18/33) exhibited a mirror difference-of-differences greater than zero, with the remainder showing no change or a decrement, consistent with the stochastic nature of crossover susceptibility. This variability aligns with emerging recognition that motor control strategies exhibit considerable individual differences reflecting diverse neural architectures and compensatory mechanisms. Recent investigations employing state-dependent interhemispheric inhibition measurements in stroke populations reveal that individual differences in motor behavior correlate with heterogeneous patterns of transcallosal communication [36]. The clinical translation of mirror-based interventions may benefit from phenotyping approaches identifying individuals most likely to demonstrate favorable responses based on baseline neurophysiological characteristics and/or task-specific motor control patterns.

Our rigorous experimental design incorporating randomized crossover structure with adequate washout periods, standardized environmental conditions, and blinded post-intervention assessments strengthens confidence in the validity of observed effects. Incorporating both age groups within identical protocol parameters enabled direct developmental comparisons that transcend the limitations of cross-sectional cohort designs. Sample size determination through formal power analysis targeting medium-sized interactions with adequate statistical power (1 − β = 0.80) provided appropriate sensitivity for detecting theoretically meaningful effects while controlling Type I error through Holm step-down correction across multiple primary outcomes.

Future investigations should (i) examine whether mirror-induced crossover attenuation persists across extended exposure durations, (ii) investigate dose–response relationships between mirror training frequency and magnitude of contralateral preservation, and (iii) evaluate generalizability to dynamic movement contexts requiring complex coordination patterns. Integrating multimodal neuroimaging approaches combining transcranial magnetic stimulation, electroencephalography, and functional near-infrared spectroscopy would elucidate temporal dynamics of cortical activation patterns accompanying mirror-induced behavioral modulation. Longitudinal designs tracking developmental trajectories of mirror responsiveness from early childhood through adolescence would clarify critical periods for sensory-motor integration maturation and inform age-specific optimization of mirror-based rehabilitation protocols.

### Practical Recommendations

Within the constraints of our sample (recreationally active soccer participants aged 10 to 36 years), practitioners implementing unilateral training protocols may consider visual feedback manipulation as a strategy for mitigating unwanted contralateral interference effects in handgrip musculature. Mirror visual feedback provides a low-cost, accessible intervention requiring minimal specialized equipment while generating large-magnitude behavioral benefits for distal upper limb musculature. Rehabilitation specialists treating asymmetrical limb disorders may integrate mirror therapy into conventional occupational therapy programs, recognizing that efficacy demonstrates muscle-selective properties favoring hand musculature over proximal shoulder-elbow complexes. Within the tested developmental range, mirror responsiveness did not differ statistically between children (aged 10–13 years) and adults (aged 19–36 years); formal equivalence testing was not conducted, and extrapolation to broader age ranges requires independent empirical verification before modification of intervention parameters can be excluded. Clinicians should acknowledge substantial inter-individual variability in mirror responsiveness, warranting individualized assessment of treatment efficacy through objective outcome monitoring rather than assuming universal benefit across all patients.

## 5. Conclusions

Mirror visual feedback attenuates crossover fatigue specifically in handgrip musculature in recreationally active individuals. The large-magnitude efficacy we observed during single-session acute exposure contrasts with previous reports suggesting dose–response relationships potentially requiring extended training durations. Our findings indicate that mirror-induced neuromodulation generates immediate countermeasure effects against contralateral performance decrements in the tested age ranges (children: 10 to 13 years; adults: 19 to 36 years). The muscle-group specificity we documented supports targeted application for distal upper limb musculature. Generalization to populations differing in age, training status, or athletic background requires empirical verification. Within our sample, mirror responsiveness did not differ statistically between children (aged 10–13 years) and adults (aged 19–36 years), indicating no statistically detectable age moderation within the tested developmental range, though formal equivalence testing would be required to support developmental invariance claims. This finding warrants replication in larger developmental cohorts spanning broader age ranges.

## Figures and Tables

**Table 1:** Anthropometric characteristics and baseline maximal voluntary isometric contraction force production stratified by age group, experimental condition, limb dominance, and muscle action domain.

Variable	Adults (*n* = 17)	Children (*n* = 16)	Cohen’s d	*p* Value
**Anthropometric Characteristics**				
Age (years)	24.64 ± 5.38	11.87 ± 0.79	3.52	<0.001
Height (cm)	177.58 ± 6.12	147.03 ± 4.82	2.84	<0.001
Body Mass (kg)	74.38 ± 6.82	45.29 ± 6.13	4.93	<0.001
Body Mass Index (kg·m^−2^)	23.61 ± 1.58	20.93 ± 1.89	1.48	<0.001
**Baseline Handgrip Force (kg)**				
Dominant—Mirror	46.78 ± 6.24	20.44 ± 4.12	4.97	<0.001
Dominant—No-Mirror	46.53 ± 6.38	20.31 ± 4.23	4.89	<0.001
Dominant—Control	46.94 ± 6.51	20.56 ± 4.07	4.86	<0.001
Non-dominant—Mirror	45.76 ± 6.18	19.84 ± 3.98	4.94	<0.001
Non-dominant—No-Mirror	45.41 ± 6.29	19.69 ± 4.14	4.82	<0.001
Non-dominant—Control	45.88 ± 6.42	19.91 ± 4.03	4.8	<0.001
**Baseline Elbow Flexion Force (kg)**				
Dominant—Mirror	39.84 ± 4.82	18.84 ± 4.23	4.53	<0.001
Dominant—No-Mirror	39.69 ± 4.71	18.71 ± 4.19	4.61	<0.001
Dominant—Control	39.92 ± 4.89	18.91 ± 4.28	4.49	<0.001
Non-dominant—Mirror	37.71 ± 4.56	18.49 ± 4.14	4.32	<0.001
Non-dominant—No-Mirror	37.54 ± 4.62	18.38 ± 4.08	4.28	<0.001
Non-dominant—Control	37.82 ± 4.69	18.56 ± 4.19	4.25	<0.001
**Baseline Elbow Extension Force (kg)**				
Dominant—Mirror	33.32 ± 4.51	14.84 ± 3.87	4.38	<0.001
Dominant—No-Mirror	33.18 ± 4.47	14.72 ± 3.94	4.43	<0.001
Dominant—Control	33.41 ± 4.58	14.91 ± 3.81	4.35	<0.001
Non-dominant—Mirror	33.01 ± 4.39	16.62 ± 3.69	4.01	<0.001
Non-dominant—No-Mirror	32.88 ± 4.44	16.51 ± 3.73	3.95	<0.001
Non-dominant—Control	33.12 ± 4.52	16.69 ± 3.64	3.98	<0.001

**Note.** Values represent mean ± standard deviation. Cohen’s d calculated using pooled standard deviation for independent-samples comparisons. Between-condition baseline equivalence verified via one-way repeated-measures ANOVA within age strata (adults: F_2,32_ = 0.62, *p* = 0.544; children: F_2,30_ = 0.78, *p* = 0.467).

**Table 2:** Pooled model-estimated marginal mean contrasts for non-dominant limb change score (Post − Pre, kg), derived from the full factorial LMM (Limb = Non-dominant stratum), pooled across age groups.

Outcome	Contrast	Estimate (kg)	95% CI	*p* Value	Cohen’s dz
Handgrip	Mirror vs. Control	0.02	[−1.15, +1.17]	0.987	0.003
Handgrip	No-Mirror vs. Control	−3.37	[−4.40, −2.35]	<0.001	−1.164
Handgrip	Mirror vs. No-Mirror	3.38	[+2.43, +4.34]	<0.001	1.255
Elbow Flexion	Mirror vs. Control	−0.40	[−1.37, +0.57]	0.405	−0.147
Elbow Flexion	No-Mirror vs. Control	−0.41	[−1.28, +0.47]	0.353	−0.164
Elbow Flexion	Mirror vs. No-Mirror	0.01	[−0.95, +0.96]	0.99	0.002
Elbow Extension	Mirror vs. Control	−0.14	[−1.36, +1.08]	0.818	−0.040
Elbow Extension	No-Mirror vs. Control	−0.11	[−1.24, +1.02]	0.845	−0.034
Elbow Extension	Mirror vs. No-Mirror	−0.03	[−0.68, +0.62]	0.925	−0.017

**Note.** Contrasts represent model-estimated marginal mean differences in non-dominant limb change score (Post − Pre), defined as within-subject difference-of-differences ([Post − Pre]_condition − [Post − Pre]_control), extracted from the full factorial LMM via the emmeans package [30] with Tukey HSD adjustment, pooled across age groups within the Limb = Non-dominant stratum. For this balanced complete dataset, these estimates are equivalent to paired *t*-tests on participant-level difference-of-differences. These pooled estimates are not equivalent to the unweighted arithmetic contrasts presented in Table 3, which are stratified by age group. Hopkins et al. [31] thresholds: small 0.20–0.60, moderate 0.60–1.20, and large 1.20–2.00.

**Table 3:** Age-stratified raw descriptive statistics: Pre-to-Post force changes, within-group pairwise comparisons, and effect sizes for non-dominant limb. Values represent observed means ± standard deviations and within-age-group contrasts. Primary hypothesis tests based on linear mixed-effects models pooled across age groups are reported in main text.

Adults (*n* = 17)								
Outcome	Condition	Pre (kg)	Post (kg)	Δ (kg)	Contrast	ΔΔ (kg)	*p* Value	Cohen’s dz
Handgrip	Control	44.12 ± 4.11	44.35 ± 4.16	0.24 ± 1.73 [−0.68, 1.15]	No−Mirror vs. Control	−4.65 [−6.35, −2.95]	<0.001	−1.45
	No-Mirror	44.50 ± 5.51	40.09 ± 6.45	−4.41 ± 2.59 [−5.79,−3.04]	Mirror vs. Control	−0.32 [−2.55, 1.91]	0.766	−0.08
	Mirror	44.38 ± 4.67	44.30 ± 5.23	−0.08 ± 3.20 [−1.78, 1.61]	Mirror vs. No−Mirror	4.33 [2.74, 5.92]	<0.001	1.44
Elbow Flexion	Control	38.96 ± 6.05	38.72 ± 5.06	−0.24 ± 2.08 [−1.34, 0.86]	No−Mirror vs. Control	−0.74 [−2.31, 0.84]	0.337	−0.25
	No-Mirror	39.10 ± 5.27	38.12 ± 4.84	−0.98 ± 1.70 [−1.88,−0.08]	Mirror vs. Control	−1.09 [−2.69, 0.51]	0.166	−0.36
	Mirror	38.12 ± 5.31	36.79 ± 4.70	−1.34 ± 2.85 [−2.85, 0.18]	Mirror vs. No−Mirror	−0.36 [−2.03, 1.31]	0.654	−0.11
Elbow Extension	Control	34.91 ± 4.54	34.46 ± 3.42	−0.44 ± 3.42 [−2.26, 1.37]	No−Mirror vs. Control	0.15 [−1.99, 2.30]	0.882	0.04
	No-Mirror	33.59 ± 3.94	33.30 ± 3.53	−0.29 ± 1.20 [−0.93, 0.35]	Mirror vs. Control	0.33 [−1.93, 2.58]	0.761	0.08
	Mirror	33.65 ± 3.77	33.54 ± 3.98	−0.11 ± 1.56 [−0.94, 0.72]	Mirror vs. No−Mirror	0.18 [−0.93, 1.28]	0.739	0.08
**Children (*n* = 16)**								
Handgrip	Control	19.26 ± 4.56	19.36 ± 4.03	0.09 ± 1.20 [−0.57, 0.76]	No−Mirror vs. Control	−2.02 [−2.86, −1.18]	<0.001	−1.32
	No-Mirror	18.69 ± 5.22	16.77 ± 4.51	−1.93 ± 1.33 [−2.66,−1.19]	Mirror vs. Control	0.36 [−0.51, 1.23]	0.397	0.23
	Mirror	19.21 ± 4.46	19.66 ± 4.89	0.45 ± 0.91 [−0.05, 0.95]	Mirror vs. No−Mirror	2.38 [1.42, 3.33]	<0.001	1.37
Elbow Flexion	Control	18.66 ± 4.73	18.54 ± 4.72	−0.12 ± 1.20 [−0.78, 0.54]	No−Mirror vs. Control	−0.06 [−0.95, 0.83]	0.895	−0.03
	No-Mirror	18.51 ± 3.81	18.34 ± 4.06	−0.17 ± 0.97 [−0.71, 0.36]	Mirror vs. Control	0.34 [−0.78, 1.45]	0.528	0.17
	Mirror	18.04 ± 4.83	18.26 ± 4.71	0.22 ± 1.53 [−0.62, 1.06]	Mirror vs. No−Mirror	0.39 [−0.66, 1.44]	0.437	0.21
Elbow Extension	Control	16.00 ± 3.99	16.07 ± 3.69	0.07 ± 0.88 [−0.41, 0.55]	No−Mirror vs. Control	−0.46 [−1.37, 0.45]	0.303	−0.28
	No-Mirror	16.22 ± 3.32	15.83 ± 3.08	−0.39 ± 1.12 [−1.00, 0.23]	Mirror vs. Control	−0.71 [−1.80, 0.39]	0.189	−0.36
	Mirror	16.76 ± 4.54	16.12 ± 4.67	−0.64 ± 1.60 [−1.52, 0.25]	Mirror vs. No−Mirror	−0.25 [−1.03, 0.53]	0.502	−0.18

**Note.** This table presents raw descriptive statistics stratified by age group to demonstrate developmental consistency of mirror effects. Primary inferential statistics (pooled linear mixed-effects model estimates) are reported in the Results text and Abstract. Values represent observed mean ± standard deviation. Pre = pre-intervention MVIC; Post = post-intervention MVIC; Δ = raw change score (Post − Pre) with 95% confidence interval calculated from within-group standard error; ΔΔ = within-age-group pairwise contrast (difference-of-differences) with 95% CI from paired *t*-tests; Cohen’s dz = within-subjects standardized effect size. Pooled model-based estimates: Mirror vs. Control +0.02 kg [−1.15, +1.17], *p* = 0.987; No-Mirror vs. Control −3.37 kg [−4.40, −2.35], *p* < 0.001; Mirror vs. No-Mirror +3.38 kg [+2.43, +4.34], *p* < 0.001. Raw within-age-group ΔΔ contrasts are unweighted paired arithmetic differences and are not directly comparable to the pooled model-estimated contrasts in Table 2. The estimand for Table 2 is the within-subject difference-of-differences in non-dominant limb change score (see Section 2.6, Materials and Methods); pooled marginal means incorporate LMM random-effects weighting across the full factorial structure.

## Data Availability

De-identified raw data, complete R analysis scripts, statistical output, and data processing changelog are publicly available in the Open Science Framework repository at https://osf.io/eumvd (https://doi.org/10.17605/OSF.IO/9VADB, (accessed on 25 February 2026)). The repository includes: (1) anonymized participant-level data in CSV format, (2) annotated R scripts reproducing all reported analyses, (3) session information documenting R version and package versions, (4) a changelog describing transcription error detection and correction with before/after statistical comparisons, and (5) a codebook defining all variables and measurement units.

## References

[1] Halperin I., Chapman D.W., Behm D.G. (2015). Non-local muscle fatigue: Effects and possible mechanisms. Eur. J. Appl. Physiol..

[2] Behm D.G., Alizadeh S., Hadjizedah Anvar S., Hanlon C., Ramsay E., Mahmoud M.M.I., Whitten J., Fisher J.P., Prieske O., Chaabene H. (2021). Non-local Muscle Fatigue Effects on Muscle Strength, Power, and Endurance in Healthy Individuals: A Systematic Review with Meta-analysis. Sports Med..

[3] Carr J.C., Bemben M.G., Stock M.S., DeFreitas J.M. (2021). Ipsilateral and contralateral responses following unimanual fatigue with and without illusionary mirror visual feedback. J. Neurophysiol..

[4] Dhahbi W. (2025). Editorial: Advancing biomechanics: Enhancing sports performance, mitigating injury risks, and optimizing athlete rehabilitation. Front. Sports Act. Living.

[5] Kavanagh J.J., Feldman M.R., Simmonds M.J. (2016). Maximal intermittent contractions of the first dorsal interosseous inhibits voluntary activation of the contralateral homologous muscle. J. Neurophysiol..

[6] Li Y., Power K.E., Marchetti P.H., Behm D.G. (2019). The effect of dominant first dorsal interosseous fatigue on the force production of a contralateral homologous and heterologous muscle. Appl. Physiol. Nutr. Metab..

[7] Gioda J., Da Silva F., Monjo F., Corcelle B., Bredin J., Piponnier E., Colson S.S. (2024). Immediate crossover fatigue after unilateral submaximal eccentric contractions of the knee flexors involves peripheral alterations and increased global perceived fatigue. PLoS ONE.

[8] Greenhouse-Tucknott A., Wrightson J.G., Raynsford M., Harrison N.A., Dekerle J. (2020). Interactions between perceptions of fatigue, effort, and affect decrease knee extensor endurance performance following upper body motor activity, independent of changes in neuromuscular function. Psychophysiology.

[9] Aboodarda S.J., Zhang C.X.Y., Sharara R., Cline M., Millet G.Y. (2019). Exercise-Induced Fatigue in One Leg Does Not Impair the Neuromuscular Performance in the Contralateral Leg but Improves the Excitability of the Ipsilateral Corticospinal Pathway. Brain Sci..

[10] Deconinck F.J., Smorenburg A.R., Benham A., Ledebt A., Feltham M.G., Savelsbergh G.J. (2015). Reflections on mirror therapy: A systematic review of the effect of mirror visual feedback on the brain. Neurorehabil. Neural Repair.

[11] Zult T., Howatson G., Kádár E.E., Farthing J.P., Hortobágyi T. (2014). Role of the mirror-neuron system in cross-education. Sports Med..

[12] Zult T., Goodall S., Thomas K., Solnik S., Hortobágyi T., Howatson G. (2016). Mirror Training Augments the Cross-education of Strength and Affects Inhibitory Paths. Med. Sci. Sports Exerc..

[13] Casamento-Moran A., Fleeman R., Chen Y.T., Kwon M., Fox E.J., Yacoubi B., Christou E.A. (2018). Neuromuscular variability and spatial accuracy in children and older adults. J. Electromyogr. Kinesiol..

[14] Ben Othman A., Behm D.G., Chaouachi A. (2018). Evidence of homologous and heterologous effects after unilateral leg training in youth. Appl. Physiol. Nutr. Metab..

[15] Ben Othman A., Chaouachi M., Makhlouf I., Farthing J.P., Granacher U., Behm D.G., Chaouachi A. (2020). Unilateral Elbow Flexion and Leg Press Training Induce Cross-Education But Not Global Training Gains in Children. Pediatr. Exerc. Sci..

[16] Ben Othman A., Hadjizadeh Anvar S., Aragão-Santos J.C., Behm D.G., Chaouachi A. (2024). Relative Cross-Education Training Effects of Male Youth Exceed Male Adults. J. Strength Cond. Res..

[17] Ben Othman A., Hadjizadeh Anvar S., Aragão-Santos J.C., Chaouachi A., Behm D.G. (2025). Age, Sex, and Training Specific Effects on Cross-Education Training. Pediatr. Exerc. Sci..

[18] Ben Othman A., Chaouachi A., Chaouachi M., Makhlouf I., Farthing J.P., Granacher U., Behm D.G. (2019). Dominant and nondominant leg press training induce similar contralateral and ipsilateral limb training adaptations with children. Appl. Physiol. Nutr. Metab..

[19] Ben Othman A., Chaouachi A., Hammami R., Chaouachi M.M., Kasmi S., Behm D.G. (2017). Evidence of nonlocal muscle fatigue in male youth. Appl. Physiol. Nutr. Metab..

[20] Turki O., Dhahbi W., Gueid S., Hmaied S., Souaifi M., Khalifa R. (2020). Dynamic Warm-Up With a Weighted Vest: Improvement of Repeated Change-of-Direction Performance in Young Male Soccer Players. Int. J. Sports Physiol. Perform..

[21] Halperin I., Aboodarda S.J., Button D.C., Andersen L.L., Behm D.G. (2014). Roller massager improves range of motion of plantar flexor muscles without subsequent decreases in force parameters. Int. J. Sports Phys. Ther..

[22] Leung M., Rantalainen T., Teo W.P., Kidgell D. (2015). Motor cortex excitability is not differentially modulated following skill and strength training. Neuroscience.

[23] Saghaei M. (2004). Random allocation software for parallel group randomized trials. BMC Med. Res. Methodol..

[24] Racinais S., Oksa J. (2010). Temperature and neuromuscular function. Scand. J. Med. Sci. Sports.

[25] Roberts H.C., Denison H.J., Martin H.J., Patel H.P., Syddall H., Cooper C., Sayer A.A. (2011). A review of the measurement of grip strength in clinical and epidemiological studies: Towards a standardised approach. Age Ageing.

[26] Stark T., Walker B., Phillips J.K., Fejer R., Beck R. (2011). Hand-held dynamometry correlation with the gold standard isokinetic dynamometry: A systematic review. PM&R.

[27] Fess F., Casanova J.S. (1992). Grip strength. Clinical Assessment Recommendations.

[28] Team R.C. (2016). R: A Language and Environment for Statistical Computing.

[29] Pinheiro J. (2011). nlme: Linear and Nonlinear Mixed Effects Models.

[30] Lenth R. (2023). emmeans: Estimated Marginal Means, Aka Least-Squares Means.

[31] Hopkins W.G., Marshall S.W., Batterham A.M., Hanin J. (2009). Progressive statistics for studies in sports medicine and exercise science. Med. Sci. Sports Exerc..

[32] Chen Y.Y., Lambert K.J.M., Madan C.R., Singhal A. (2021). Mu oscillations and motor imagery performance: A reflection of intra-individual success, not inter-individual ability. Hum. Mov. Sci..

[33] Gonzalez-Santos J., Soto-Camara R., Rodriguez-Fernández P., Jimenez-Barrios M., Gonzalez-Bernal J., Collazo-Riobo C., Jahouh M., Bravo-Anguiano Y., Trejo-Gabriel-Galan J.M. (2020). Effects of home-based mirror therapy and cognitive therapeutic exercise on the improvement of the upper extremity functions in patients with severe hemiparesis after a stroke: A protocol for a pilot randomised clinical trial. BMJ Open.

[34] Sydnor V.J., Larsen B., Bassett D.S., Alexander-Bloch A., Fair D.A., Liston C., Mackey A.P., Milham M.P., Pines A., Roalf D.R. (2021). Neurodevelopment of the association cortices: Patterns, mechanisms, and implications for psychopathology. Neuron.

[35] Siddique U., Frazer A.K., Tallent J., Hayman O., Ahtiainen J.P., Avela J., Akalu Y., Rostami M., Uribe S., Walker S. (2025). A Pilot Mixed-Design Study of Cross-Education-Related Strength Gains and Reticulospinal Involvement in Young and Older Adults. Scand. J. Med. Sci. Sports.

[36] Mirdamadi J.L., Xu J., Arevalo-Alas K.M., Kam L.K., Borich M.R. (2023). State-dependent interhemispheric inhibition reveals individual differences in motor behavior in chronic stroke. Clin. Neurophysiol..

